# Multidetector-row computed tomography of thoracic aortic anomalies in dogs and cats: Patent ductus arteriosus and vascular rings

**DOI:** 10.1186/1746-6148-7-57

**Published:** 2011-09-23

**Authors:** Christiane R Henjes, Ingo Nolte, Patrick Wefstaedt

**Affiliations:** 1 Small Animal Clinic, University of Veterinary Medicine Hannover, Foundation, Hannover, Germany

## Abstract

**Background:**

Diagnosis of extracardiac intrathoracic vascular anomalies is of clinical importance, but remains challenging. Traditional imaging modalities, such as radiography, echocardiography, and angiography, are inherently limited by the difficulties of a 2-dimensional approach to a 3-dimensional object. We postulated that accurate characterization of malformations of the aorta would benefit from 3-dimensional assessment. Therefore, multidetector-row computed tomography (MDCT) was chosen as a 3-dimensional, new, and noninvasive imaging technique. The purpose of this study was to evaluate patients with 2 common diseases of the intrathoracic aorta, either patent ductus arteriosus or vascular ring anomaly, by contrast-enhanced 64-row computed tomography.

**Results:**

Electrocardiography (ECG)-gated and thoracic nongated MDCT images were reviewed in identified cases of either a patent ductus arteriosus or vascular ring anomaly. Ductal size and morphology were determined in 6 dogs that underwent ECG-gated MDCT. Vascular ring anomalies were characterized in 7 dogs and 3 cats by ECG-gated MDCT or by a nongated thoracic standard protocol.

Cardiac ECG-gated MDCT clearly displayed the morphology, length, and caliber of the patent ductus arteriosus in 6 affected dogs. Persistent right aortic arch was identified in 10 animals, 8 of which showed a coexisting aberrant left subclavian artery. A mild dilation of the proximal portion of the aberrant subclavian artery near its origin of the aorta was present in 4 dogs, and a diverticulum analogous to the human Kommerell's diverticulum was present in 2 cats.

**Conclusions:**

Contrast-enhanced MDCT imaging of thoracic anomalies gives valuable information about the exact aortic arch configuration. Furthermore, MDCT was able to characterize the vascular branching patterns in dogs and cats with a persistent right aortic arch and the morphology and size of the patent ductus arteriosus in affected dogs. This additional information can be of help with regard to improved diagnoses of thoracic anomalies and the planning of surgical interventions.

## Background

Patent ductus arteriosus (PDA) and persistent right aortic arch are 2 of the most common vascular anomalies of the thoracic aorta [1-3]. Imaging of extracardiac vascular structures in the thorax is challenging. Radiography, echocardiography, and angiography are the most frequently used diagnostic imaging modalities for patients with suspected cardiovascular diseases; however, each of these modalities is inherently compromised by difficulties of a 2-dimensional approach to a 3-dimensional object. Magnetic resonance imaging and multidetector-row computed tomography (MDCT) are 3-dimensional, noninvasive modalities. Both have proven their capabilities for the diagnosis of PDA and persistent right aortic arch in human medicine, while reports about such investigations are rare in veterinary medicine [4-10]. However, magnetic resonance imaging has the drawback of a long image acquisition time and high cost of examination.

The ductus arteriosus is a physiological vascular structure during fetal development which normally obliterates shortly after birth. If this process fails, the ductus remains open, resulting in an unintended continuous blood flow between the pulmonary artery and aorta. This condition is usually diagnosed within the first few months after birth and requires surgical intervention to prevent the development of left-sided congestive heart failure [11,12]. Treatment options include different surgical ligation techniques, as well as transcatheter embolization by means of various occlusion devices [13-18]. In cases of transcatheter occlusion, knowledge about the PDA morphology and dimensions is essential, and must be acquired prior to interventional occlusion with a catheter-delivered device [19].

Transthoracic echocardiography is the diagnostic technique of choice for the initial evaluation of dogs with suspected congenital heart diseases. The advantages of echocardiography include noninvasiveness, high accessibility, and low cost. However, this diagnostic modality is limited in its ability to depict extracardiac vascular structures, its small acoustic window, and its operator dependence. In addition, the accuracy and reliability of ductal measurements by transthoracic echocardiography have been questioned [20,21]. Angiocardiography allows direct visualization of the vascular and cardiac system, and is usually performed to determine the size and shape of the PDA immediately before transcatheter occlusion. However, in veterinary medicine, angiocardiography is commonly performed as monoplane fluoroscopy, which is seen as one potential source of inaccuracy [19,22].

The most common type of a vascular ring anomaly in dogs is a persistent right aortic arch with a left ductus arteriosus or ligamentum arteriosum [2,23]. A less common malformation is an aberrant left subclavian artery, which may occur in conjunction with a persistent right aortic arch or alone. A presumptive diagnosis of a vascular ring anomaly is commonly made from the history of the patient and results of the clinical and radiographic examinations. Generally, the most common clinical sign referring to a vascular ring anomaly is regurgitation, and it occurs in puppies or kittens when they start to eat solid food at the time of weaning. Affected animals are often thin and smaller than their littermates, and some are dyspneic because of aspiration pneumonia. Lateral survey and contrast radiographs of these animals commonly show esophageal constriction at the heart base and precardial esophageal dilation. Ventrodorsal radiographs show a leftward curvature of the trachea [23]. These characteristic radiographic findings are normally indicative of a persistent right aortic arch. Thus, further diagnostic imaging of vascular ring anatomy before surgery is rarely performed, and definitive diagnosis is achieved by surgical exploration. Most vascular ring anomalies are well managed by a left intercostal approach. However, even though persistent right aortic arch accounts for approximately 95% of vascular ring anomalies [23], less frequent anomalies can coexist, which usually cannot be visualized by radiographic examinations. Of special interest are presurgical diagnoses of malformations of the aortic arch and its branching that lead to vascular ring anomalies. Several of these anomalies, such as patent right ductus arteriosus, aberrant right subclavian artery, and some forms of double aortic arches, may necessitate a right intercostal approach [3]. Therefore, some authors recommend an additional angiographic examination before surgery [24,25]. In contrast, other authors have doubts about the use of angiography in these cases because the examination of aortic arch vessels has been proven difficult, even with biplane angiography, because of the 3-dimensional nature of vascular anomalies [23].

The recent introduction of MDCT in cardiovascular imaging achieved advantages such as rapid acquisition time, high spatial resolution, and acceptable temporal resolution. In the last decade, computed tomography has been increasingly used for the assessment of vascular anomalies such as portosystemic shunts and arterioportal fistulae [26,27]. However, computed tomography angiography of the intrathoracic vessels has been used in only a few cases [4,5,28]. Gating MDCT thoracic aorta studies to the cardiac cycle by electrocardiography (ECG) has been shown to produce significantly fewer motion artifacts than does a standard nongated acquisition protocol [29]. Because MDCT is a real, 3-dimensional imaging modality, it may overcome some of the aforementioned problems of radiography, echocardiography, and angiography during examination of the heart and adjacent structures.

The purpose of this study was to demonstrate that MDCT can provide detailed images of the anatomy of PDA and identify persistent right aortic arch in affected dogs and cats.

## Methods

Cardiac ECG-gated and thoracic nongated MDCT images were reviewed in identified cases of either PDA or vascular ring anomaly. All examinations were undertaken as part of clinical practice, and patient owners gave written informed consent before MDCT imaging was carried out. Examinations by MDCT from April 2008 to October 2010 were reviewed. Six dogs with confirmed diagnoses of PDA and 7 dogs and 3 cats with vascular ring anomaly were included in this study. The 6 dogs with PDA and 4 dogs and 1 cat with persistent right aortic arch underwent ECG-gated cardiac MDCT. The remaining 3 dogs and 2 cats with persistent right aortic arch were examined with a standard MDCT thoracic protocol.

Anesthesia was induced using conventional techniques according to the preference of the consulting anesthetist. In all cases, an endotracheal tube was placed and anesthesia was maintained with 1.5 vol % inspiratory isoflurane concentration in an air and oxygen (50%) mixture by use of mechanical ventilation.

### MDCT protocol

Contrast-enhanced MDCT examinations were carried out using a 64-detector-row computed tomography system (Brilliance 64; Philips, Netherlands). Patients were placed in the supine position, ECG leads were attached to the paws, and ECG was recorded simultaneously during spiral MDCT examination. Scans were obtained during apnea with a collimation of 64 × 0.625 mm, table pitch of 0.20, tube voltage 120 kV, tube current of 400 mA, and tube rotation time of 0.4 sec.

In cases of the nongated thoracic standard MDCT protocol, patients were placed in the prone position. Scans were obtained during apnea as follows: collimation of 64 × 0.625 mm, table pitch of 1.08, tube voltage of 120 kV, tube current of 315 mA, and tube rotation time of 0.5 sec.

All patients received 3 mL/kg body weight of the contrast medium iobitridol (Xenetix 350; Guerbet, Germany) intravenously by a peripheral vein. Because a minimal applicable volume of 10 mL was required by means of a power injector (Vistron CT Injection System; Medrad, USA), contrast media for animals weighing < 3.3 kg was diluted with 0.9% saline solution until a volume of 10 mL was achieved. The flow rate was adapted to the patient weight. Contrast medium was administered over a minimum of 20 sec at a maximal flow rate of 3 mL/sec. For the purpose of automatic detection of contrast medium arrival, the region of interest was defined in the ascending or proximal descending aorta. In cases of ECG-gated cardiac MDCT, the scan was performed automatically as soon as a predefined threshold of 110 Hounsfield units was exceeded. In cases of nongated thoracic standard MDCT, bolus tracking was started 20 sec after contrast media administration. The scan automatically started 25 sec after a threshold of 110 Hounsfield units was exceeded.

Image series of ECG-gated cardiac MDCT scans were reconstructed by a multisector reconstruction algorithm at multiple phases with increments of 10% of the R-R interval, covering the whole cardiac cycle. The data set with the least amount of motion artifact was chosen for further postprocessing.

In all cases, diagnosis of PDA and persistent right aortic arch was confirmed by surgical situs. Ligation and transection of the PDA and ligamentum arteriosum, respectively, was performed. In cases of persistent right aortic arch, visual examination and palpation verified whether compression of the esophagus, possibly caused by an aberrant left subclavian artery, still existed after ligation and division of the ligamentum arteriosum.

### PDA analysis

**Figure 1:**
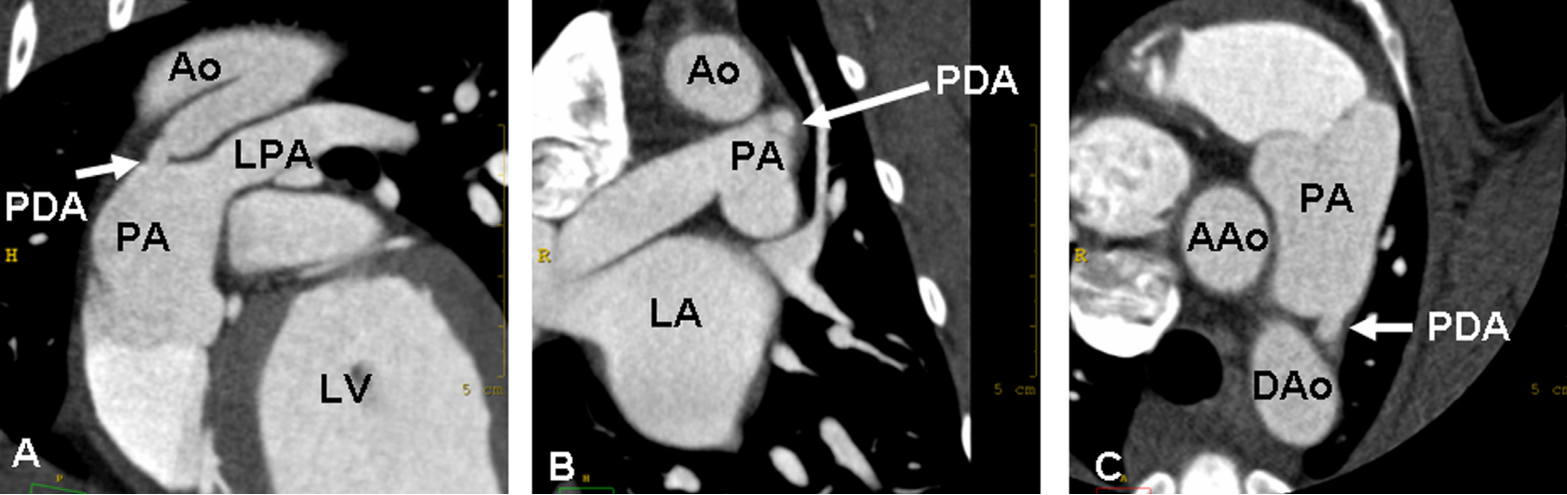
**Patent ductus arteriosus in a border collie**. MPRs of retrospectively ECG-gated cardiac MDCT show the narrowest portion of the duct in oblique sagittal (A), dorsal (B), and transversal (C) planes. Ao = aorta, AAo = ascending aorta, Dao = descending aorta, PA = pulmonic artery, LPA = left pulmonic artery, LA = left atrium, LV = left ventricle.

**Figure 2:**
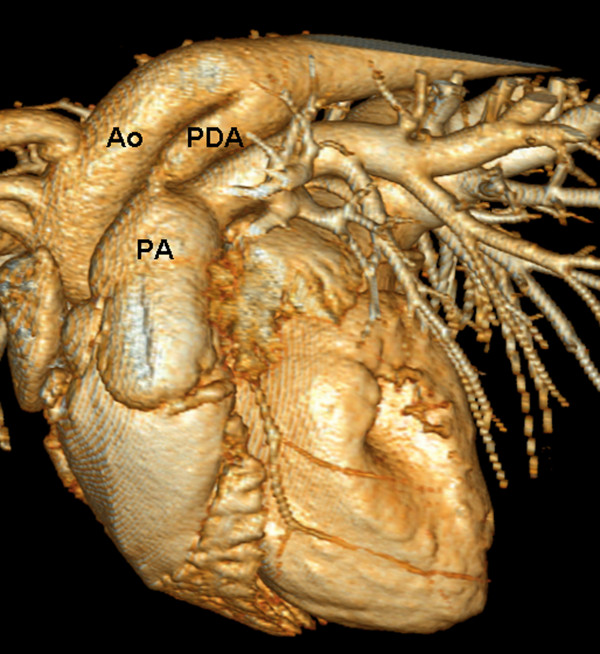
**Volume-rendered 3-dimensional MDCT reconstruction of a patent ductus arteriosus**. The patent ductus arteriosus (PDA) connects the descending aorta dorsally to the main pulmonary artery. Ao = aorta, PA = pulmonary artery.

Multiplanar reformations (MPR) were created according to the anatomical transversal, sagittal, and dorsal directions of the PDA. The smallest ductal diameter was measured in these views (Figure 1). Length and width of the PDA were measured in the sagittal plane. All dimensions were measured precisely to 0.1 mm. Sagittal MPRs and 3-dimensional volume-rendered displays were used for morphologic classification of PDAs according to the description by Miller et al. (Figures 1 and 2) [19].

### Vascular ring anomaly analysis

Images were evaluated in anatomical sagittal and transversal MPRs and in a 3-dimensional volume-rendered display. All images with diagnosed vascular ring anomalies were reviewed for the presence of a right aortic arch, branching of the common carotid arteries, and the coexistence of an aberrant left subclavian artery, double aortic arch, persistent left vena cava cranialis, and PDA. In addition, the presence of dilation of the proximal portion of an aberrant subclavian artery was noted in analogy to Kommerell's diverticulum described for humans [30]. Therefore, a diverticulum was considered present when measurement of the diameter of the subclavian artery near its origin from the aortic arch was at least twice the size of its more distal diameter [30].

## Results

### PDA

The median age of the examined dogs at the time of MDCT examination (2 Bolonka Zwetnas, 1 dachshund, 1 border collie, 1 Labrador retriever, and 1 Yorkshire terrier) was 8.5 months (range, 3-54 months), and the median weight was 3.6 kg. In all 6 dogs, PDAs were identified as a vascular structure connecting the distal portion of the aortic arch with the main pulmonary artery (Figure 1). The median ductal ampulla width was 7.7 mm (range, 5.0-12.7 mm), and the median ductal length was 12.1 mm (range, 8.5-22.4). The median minimal ductal diameter was 4.00 mm (range, 2.3-8.5 mm), 4.55 mm (range, 3.6-8.0 mm), and 4.05 mm (range, 2.5-7.0 mm) in the transversal, dorsal, and sagittal planes, respectively. All dogs showed a PDA corresponding to a classification of IIA according to the description by Miller et al. (Table 1) [19].

**Table 1:** Characteristics of Patent Ductus Arteriosus in 6 patients as determined with ECG-gated MDCT

Patients data		Characteristics of Duct					
**body weight (kg/lb)**	**age (mo)**	**minmal ductal diameter (mm)**			**ductal width (mm)**	**ductal length (mm)**	**morphologic classification**
		**transversal**	**dorsal**	**sagittal**			

3.5/7.7	53	4.7	4.5	4.8	6.6	14.0	IIA
3.3/7.3	5	3.8	4.6	3.8	8.8	8.8	IIA
17.0/37.5	54	4.2	4.9	4.3	8.9	22.4	IIA
3.7/8.2	7	3.0	3.6	2.5	5.0	12.2	IIA
9.0/19.8	3	8.5	8.0	7.0	12.7	12.0	IIA
3.6/7.9	10	2.3	3.8	2.5	5.2	8.5	IIA

### Vascular ring anomaly

The median age of the 7 dogs with vascular ring anatomy (3 German pinschers, all from the same breeder), 1 German mastiff, 1 Labrador retriever, 1 Weimaraner, and 1 mongrel dog) was 3 months (range, 2-20 months), and the median weight was 7.1 kg (range, 4.5-54 kg). Two European shorthair cats and a Thai Siamese cat were examined; their median age was 10 months (range, 3-10 months), and their median weight was 1.6 kg (range, 1.2-3.3 kg).

A right aortic arch was present in all 10 animals. A physiological truncus brachiocephalicus and normal left subclavian artery was present in only 1 cat, whereas the great arteries of the other 9 animals showed the following different patterns of branching (Table 2):

**Table 2:** Vascular ring anomalies in 10 animals

	dogs	cats
persistent right aortic arch	7	3

aberrant left subclavian artery	6	2

truncus brachiocephalicus	0	1

bicarotid trunk	5	1

separate branching of the common carotid arteries and subclavian arteries from the aorta	1	1

origin of the comon carotid arteries and the left subclavian artery at the same level	1	0

mild dilation of the aberrant left subclavian artery	4	0

Kommerell's diverticulum	0	2

**Figure 3:**
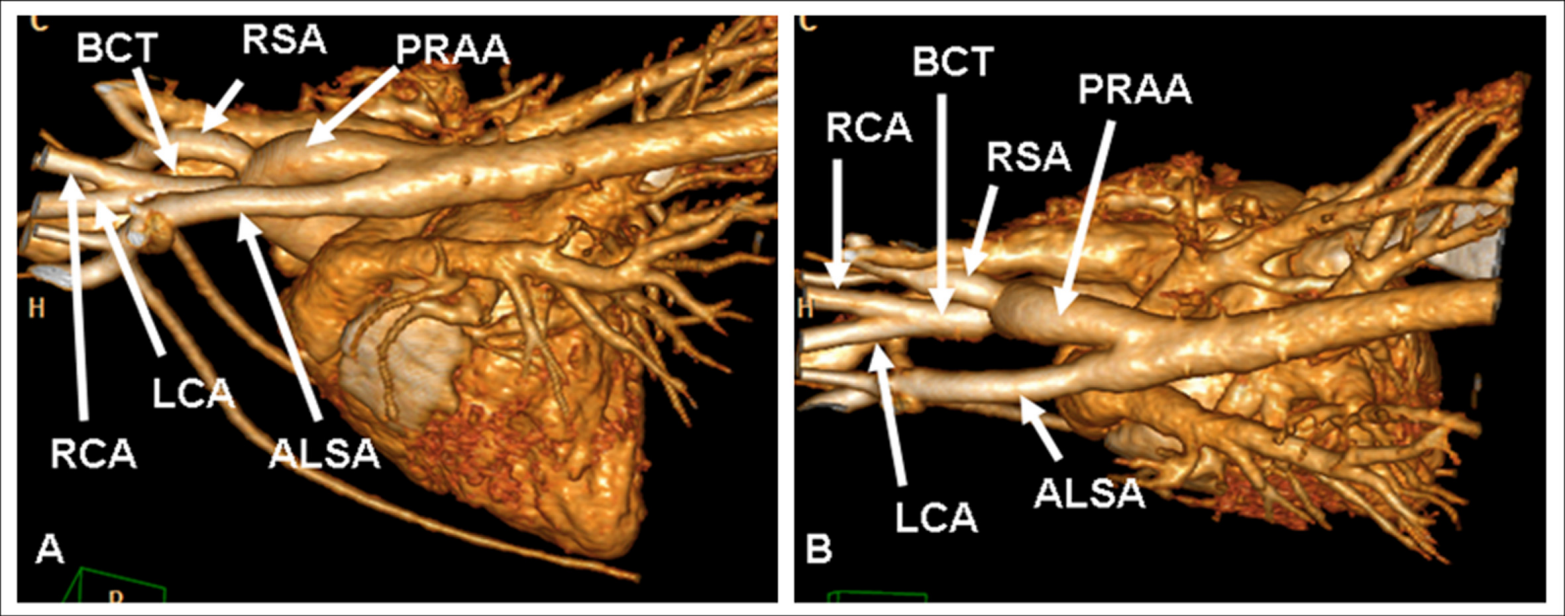
**Persistent right aortic arch with aberrant left subclavian artery and bicarotid trunk in a German pinscher**. Left lateral (A) and dorsoventral volume (B) volume-rendered 3-dimensional MDCT images. Volume-rendered images show an aberrant left subclavian artery (ALSA) with mild dilation at its origin. The bicarotid trunk arises as the first branch from the aortic arch, which is followed by the right subclavian artery (RSA) and the ALSA, which arises from the proximal part of the descending aorta. LCA = left carotid artery, RCA = right carotid artery.

1) The bicarotid trunk, right subclavian artery, and aberrant left subclavian artery successively branched from a persistent right aortic arch in 5 dogs and 1 cat (Figure 3).

2) The left carotid artery, right carotid artery, right subclavian artery, and aberrant left subclavian artery arose in order from a persistent right aortic arch in 1 dog and 1 cat.

3) The left subclavian artery and left and right carotid arteries branched from a persistent right aortic arch at the same level in 1 dog. The right subclavian artery arose some centimeters distal to this origin from the persistent right aortic arch. The left subclavian artery did not run retroesophageally and was therefore not counted as an aberrant left subclavian artery.

**Figure 4:**
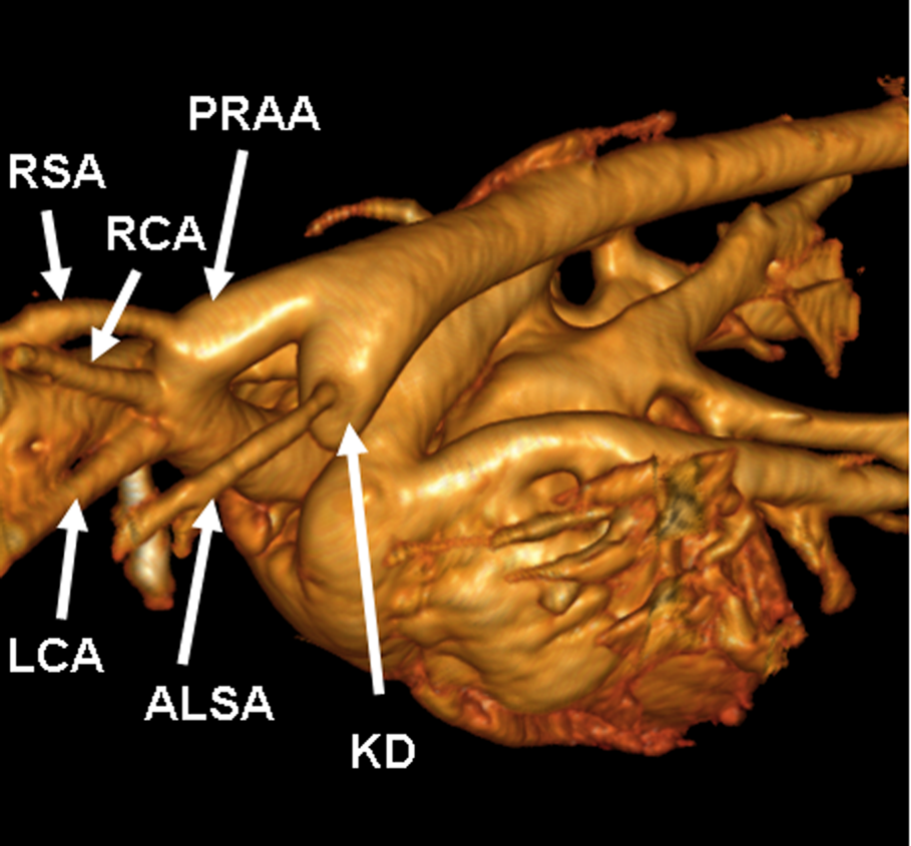
**Kommerell's diverticulum in a cat**. Volume-rendered 3-dimensional MDCT reconstruction of a persistent right aortic arch (PRAA) with aberrant left subclavian artery (ALSA) showing Kommerell's diverticulum (KD) at its origin in a European short hair. LCA = left carotid artery, RCA = right carotid artery, RSA = right subclavian artery.

All aberrant left subclavian arteries arose from the left side of the distal aortic arch or proximal descending aorta. None of the examined animals with vascular ring anomaly showed a double aortic arch, persistent left vena cava, or PDA. In 4 dogs, a mild dilation of the proximal portion of the aberrant left subclavian artery near its origin of the aorta was present, and 2 cats showed Kommerell's diverticulum (Figure 4).

Esophageal compression was best displayed in transverse MPRs and was present in all animals. Two-dimensional transversal and sagittal images were found best suitable to demonstrate the spatial relationships of the aorta with adjacent organs. All ECG-gated MDCT scans, and all but 1 examination by the thoracic standard protocol were of excellent imaging quality.

**Figure 5:**
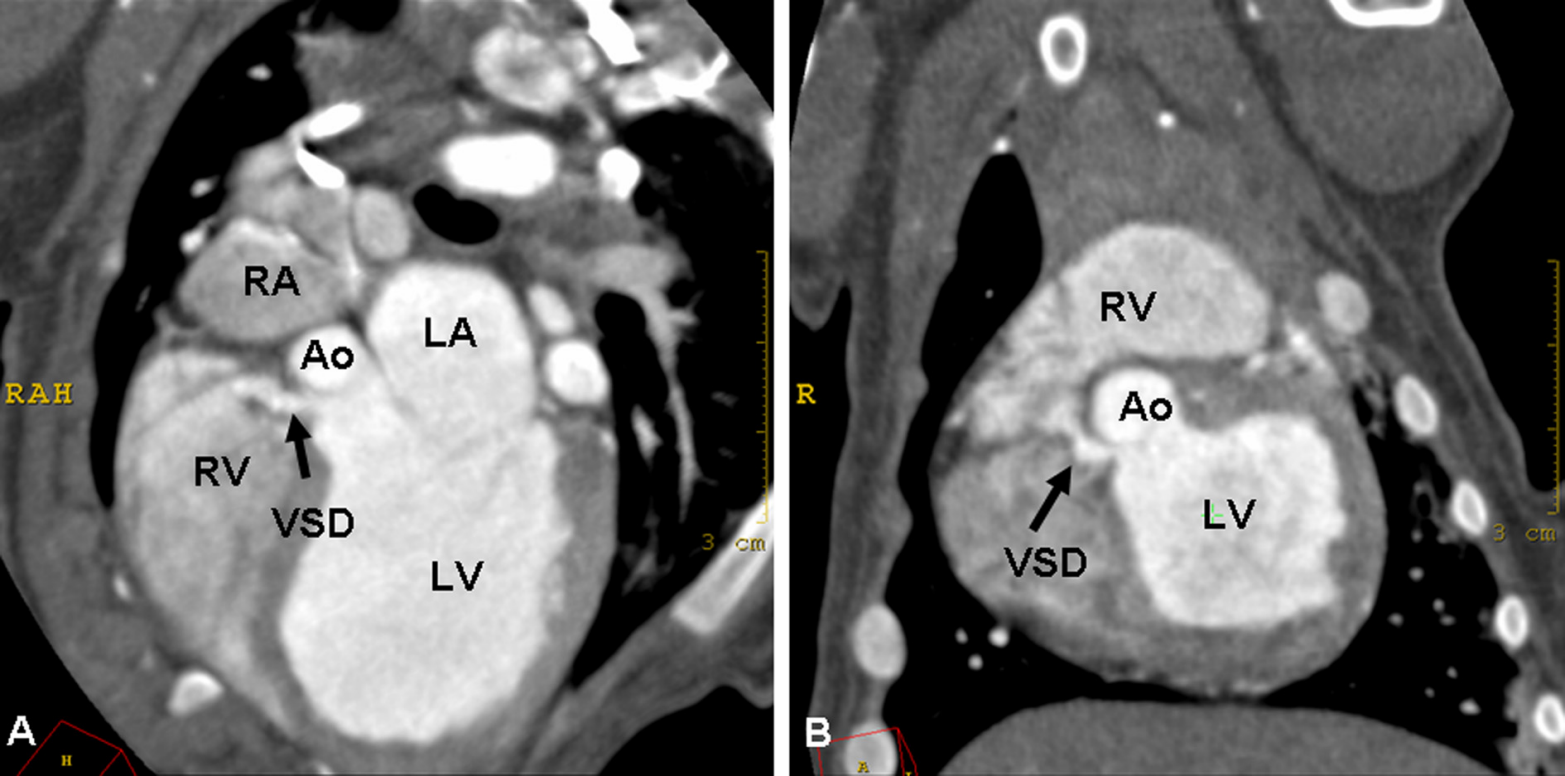
**Ventricular septal defect in a dog**. Oblique transversal (A) and dorsal (B) MPRs of contrast-enhanced ECG-gated cardiac MDCT images. The images show the ventricular septal defect (VSD) as a small contrast-enhanced connection between the right ventricle (RV) and the left ventricle (LV). LA = left atrium, RA = right atrium, Ao = aorta.

Two dogs (German pinschers) with a persistent right aortic arch showed an additional ventricular septal defect. Diagnosis was made by echocardiography, and the ventricular septal defect was demonstrated as a connection between the left and right ventricles in 1 dog imaged by ECG-gated MDCT (Figure 5). The second dog was examined by the thoracic standard MDCT protocol, and, in contrast, the connection was not visible.

## Discussion

### PDA

In our study, ECG-gated MDCT allowed an accurate diagnosis of a PDA. Furthermore, ductal size and morphologic type could be determined according to the angiographic classifications originally described by Miller et al. [19]. In the present study, MPRs and 3-dimensional volume-rendering images were used to demonstrate the presence of PDA. In particular, oblique sagittal MPR images and 3-dimensional volume-rendering images were found to be best suitable for diagnosis of PDA.

Oblique sagittal, dorsal, and transverse MPRs were created for the measurement of minimal ductal size. Consequently, we were able to examine the 3-dimensional morphology of the PDA, which is generally not possible with angiographic or echocardiographic evaluation [19]. However, morphologic assessment by angiocardiography and echocardiography were not performed. Furthermore, 3-dimensional echocardiography might be another imaging modality capable for defining the 3-dimensional anatomy of a PDA. Therefore, further studies are necessary to compare these image modalities with each other and to evaluate whether an MDCT examination may improve the accuracy of measurements and alter the treatment of affected animals.

Saunders et al. carried out transesophageal echocardiographic investigations and described the PDA ampulla in short-axis orientation as an elliptical rather than circular structure [31]. This is in accordance with our findings. Besides the ampulla itself, the measurements of the minimal ductal orifice in all 3 perpendicular views showed small deviations, indicating that it might not always have a perfectly circular appearance.

A disadvantage of MDCT in animals is the necessity of anesthesia. In the case of PDA, affected dogs are potentially compromised, making additional anesthesia for MDCT examination undesirable. However, because MDCT is a fast image acquisition modality, surgical correction or transcatheter closure can be accomplished in the same anesthetic episode. Furthermore, in the case of transcatheter PDA closure, a previous cardiac MDCT evaluation might be suitable to reduce the required number of contrast medium injections and the duration of angiography because size and morphology of PDA can be evaluated before angiography is carried out. This will reduce radiation exposure to the surgeon and staff. Furthermore, previous MDCT examination will decrease angiography time, whereas the overall anesthesia time might increase by a few minutes. Bearing in mind that not every PDA is suitable for transcatheter device occlusion, angiocardiographic examinations of these patients can be avoided by cardiac MDCT examination. This makes a prolonged anesthesia reasonable.

### Vascular rings

Vascular rings are developmental anomalies of the main thoracic arteries and most commonly cause esophageal constriction. Several types of vascular ring anomalies have been reported in dogs and cats [2,3]. In this study, we determined different types of vascular ring anomalies in dogs and cats by contrast-enhanced ECG-gated computed tomography and by MDCT with a thoracic standard protocol. A persistent right aortic arch was present in all examined cases, and an aberrant left subclavian artery was most commonly observed. In fact, only 1 cat showed no further coexisting vascular anomaly. An aberrant left subclavian artery in conjunction with a persistent right aortic arch has been previously reported, although this coexistence was less often reported than was observed in the present study [23]. Further commonly reported anomalies coexisting with vascular ring anomalies are PDA and persistent left cranial vena cava. However, none of these anomalies were recognized in this study.

In veterinary medicine, some authors recommend ligation of an aberrant left subclavian artery because it may result in a dorsal constriction as the artery crosses the esophagus [3]. In this study, only ligation and transection of the ligamentum arteriosum were performed because no remaining constriction of the esophagus was observed in any patient after ligamentum division. The fact that an aberrant subclavian artery did not lead to an esophageal compression may be explained by the distal origin of the aberrant subclavian artery in all cases. While the persistent right aortic arch lies at the right of the midline, the proximal part of the descending aorta already approaches the midline. In all cases, the left subclavian artery arose from the left side of the distal part of the aortic arch or the proximal part of the descending aorta. Therefore, its origin was quite dorsal and just beside the midline, leaving enough space for the esophagus after ligamentum division.

One main finding of this study was a diverticulum at the origin of the aberrant left subclavian artery in 2 cats. Similar findings are well described in human medicine and are defined as Kommerell's diverticulum. Kommerell's diverticulum is a dilation of the proximal portion of an aberrant subclavian artery near its origin from the aorta and is expected to be a remnant of the left fourth aortic arch [30]. This diverticulum has been shown to compress the trachea and esophagus independently from the effects of the right aortic arch and left ligamentum arteriosum in children with vascular rings [32]. Furthermore, it can cause recurrent symptoms in human patients if only division of the ligamentum arteriosusm is performed [32]. Therefore, ligation of the aberrant left subclavian artery and resection of Kommerell's diverticulum or resection of Kommerell's diverticulum and reanastomosis of the aberrant left subclavian artery into the left carotid artery is recommended by some authors in human medicine [32]. In addition, 4 dogs showed a mild dilation of the aberrant left subclavian artery at its aortic origin. Analogous conditions were described in a case report of 2 dogs in veterinary medicine [25]. However, to the authors' knowledge, there have been no previous reports of Kommerell's diverticulum in cats. The clinical relevance of Kommerell's diverticulum in dogs and cats remains unclear. Complications comparable with those in human medicine are possible, but not yet investigated. Further examinations of vascular ring anomalies by MDCT might be helpful to identify Kommerell's diverticulum more often and to evaluate its clinical relevance.

In several reported cases, coexisting or rare vascular ring anomalies were not identified by presurgical angiography or even by surgical exploration [24,33]. This is not unexpected, because the identification of rare or coexisting ring anomalies, such as an aberrant subclavian artery, requires accurate exploration and specific dissection of the region dorsal to the esophagus [23]. Preoperative MDCT angiography can confirm the suspected diagnosis of coexisting vascular anomalies and can help in surgical treatment planning, making only minimal dissection necessary in truly affected animals.

In the case of suspected vascular ring anomaly, 2 different MDCT imaging modalities were performed. Whether a nongated thoracic MDCT protocol or an ECG-gated cardiac protocol was applied depended on the radiologist in charge. However, in 1 case, an ECG-gated MDCT was planned but could not be performed because the heart rate was greater than 130 beats per minute, which exceeded the limitations of ECG-gating in our CT scanner. Therefore, a nongated thoracic standard MDCT protocol was performed. Although coexisting cardiac anomalies are rare, 2 dogs in our study showed a ventricular septal defect. Diagnosis was made by echocardiography, and we were able to demonstrate the connection between the left and right ventricles with ECG-gated MDCT as well (Figure 5). However, the ventricular septal defect was not visible on the images taken by the nongated thoracic standard protocol. Because of the rapid heart movement, motion artifacts caused blurring of the ventricular cavities and interventricular septum and masked the connection between the left and right ventricles. Although ECG-gated images were able to demonstrate the ventricular septal defect, echocardiography appeared to be a more suitable imaging modality for the evaluation of simple lesions of the cardiac septum in our analysis. In particular, color Doppler flow echocardiography allowed the evaluation of cardiac flow across the ventricular septal defects.

Although transverse images are sufficient for the evaluation of vascular ring anomalies, multiplanar and 3-dimensional volume-rendered images facilitate the evaluation of cardiovascular anatomy. In particular, interactive rotation of 3-dimensional volume-rendered images was employed for the optimal understanding of spatial relationships. Although 3-dimensional image modalities are of great value, they remain time-consuming. Therefore, image acquisition and surgery should be performed on different days to avoid considerably prolonged anesthesia. Bearing in mind that vascular ring anomalies do not impair cardiac performance, we consider additional anesthesia for MDCT imaging reasonable.

Former major technical disadvantages of computed tomography, namely single slice scan acquisition and poor temporal resolution, have been greatly reduced by the introduction of MDCT combined with ECG-gating. However, some limitations of computed tomography remain and must be considered. MDCT is less available and more expensive than traditional imaging methods (radiography, angiography, and echocardiography). Furthermore, the ECG-gating technique is restricted to a maximal heart rate. However, the MDCT technique is especially advantageous in the diagnosis of congenital anomalies of the thoracic aorta. The large field of view and MPRs in any desired plane provide good visualization of the complex anatomy of these vascular anomalies and identify associated cardiac malformations. Three-dimensional images by MDCT are suitable to show the anatomy of aortic anomalies (PDA or persistent right aortic arch) and the spatial relationship of adjacent structures and can therefore provide valuable information for surgeons. Even without gating, motion artifacts were not relevant at the level of the aortic arch and its branching, and image quality was judged excellent in all but 1 case. Contrast enhancement of the aorta and great arteries in this cat compared with the other examinations was less, and delineation of the vascular system was harder to achieve. A possible reason for the impaired image quality is the low animal weight of 1.6 kg. However, images of another cat weighing 1.3 kg were acquired by the ECG-gated MDCT protocol and were of excellent image quality. A high dose of contrast medium was used in this study to achieve distinct contrast enhancement of the arterial system and cardiac chambers simultaneously. Therefore, application of the thoracic protocol, which leads to greater motion artifacts than do ECG-gated images, or timing of the contrast medium application is more likely to be of importance.

Limitations of this study include patient variability within a small sample size and the retrospective study design. Another drawback of this study is the lack of comparative data to a "gold-standard," such as catheter-based angiography, for direct comparison in the sizing of the ductus arteriosus.

## Conclusions

In conclusion, contrast-enhanced MDCT is a reliable and noninvasive tool for diagnosing PDA and persistent right aortic arch. This modality allows an accurate anatomic diagnosis of vascular anomalies. It provides valuable information about the exact aortic arch configuration and vascular branching patterns in cases of persistent right aortic arch and about the ductal morphology, length, and caliber in cases of PDA.

## Authors' contributions

CRH collected and analyzed the data, wrote the manuscript, and made the figures. CRH and PW designed the study. PW and IN helped to draft the manuscript. All authors read and approved the final manuscript.
